# Inhibition of Electrical Activity by Retroviral Infection with Kir2.1 Transgenes Disrupts Electrical Differentiation of Motoneurons

**DOI:** 10.1371/journal.pone.0002971

**Published:** 2008-08-13

**Authors:** Yone Jung Yoon, Hisashi Kominami, Thomas Trimarchi, Miguel Martin-Caraballo

**Affiliations:** Department of Biology, University of Vermont, Burlington, Vermont, United States of America; Emory University, United States of America

## Abstract

Network-driven spontaneous electrical activity in the chicken spinal cord regulates a variety of developmental processes including neuronal differentiation and formation of neuromuscular structures. In this study we have examined the effect of chronic inhibition of spinal cord activity on motoneuron survival and differentiation. Early spinal cord activity in chick embryos was blocked using an avian replication-competent retroviral vector RCASBP (B) carrying the inward rectifier potassium channel Kir2.1. Chicken embryos were infected with one of the following constructs: RCASBP(B), RCASBP(B)-Kir2.1, or RCASBP(B)-GFP. Infection of chicken embryos at E2 resulted in widespread expression of the viral protein marker p27 gag throughout the spinal cord. Electrophysiological recordings revealed the presence of functional Kir2.1 channels in RCASBP(B)-Kir2.1 but not in RCASBP(B)-infected embryos. Kir2.1 expression significantly reduced the generation of spontaneous motor movements in chicken embryos developing *in ovo*. Suppression of spontaneous electrical activity was not due to a reduction in the number of surviving motoneurons or the number of synapses in hindlimb muscle tissue. Disruption of the normal pattern of activity in chicken embryos resulted in a significant downregulation in the functional expression of large-conductance Ca^2+^-dependent K^+^ channels. Reduction of spinal cord activity also generates a significant acceleration in the inactivation rate of A-type K^+^ currents without any significant change in current density. Kir2.1 expression did not affect the expression of voltage-gated Na^+^ channels or cell capacitance. These experiments demonstrate that chronic inhibition of chicken spinal cord activity causes a significant change in the electrical properties of developing motoneurons.

## Introduction

Electrical activity plays an important role in regulating growth and development of the nervous system. For example, ongoing electrical activity appears to regulate neuronal survival, axon outgrowth, neurotransmitter expression, and electrical differentiation of developing neurons [1]–[4]. Spontaneous electrical activity is an early feature of the chicken spinal cord. This activity appears to be crucial for the development of spinal cord neurons and for proper maturation of the neuromuscular system [2], [5]–[7]. Bursts of electrical activity can be recorded from specific motor nerves at embryonic day (E) 4 (corresponding to stage 24), 48 hrs prior to target innervation [8]. Spontaneous electrical activity underlies the generation of rhythmic motor movements (or limb movements) in the developing chicken embryo following the establishment of synaptic connections between nerve terminals and muscle fibers at E6 (corresponding to stage 28–29) [9]. Transection of the spinal cord does not prevent the generation of early activity suggesting that it originates from intrinsic spinal cord networks located in the ventral cord [10]–[12]. In the chicken spinal cord, the neuronal circuits that generate spontaneous activity at early stages of development (between E4–E6) rely solely on cholinergic and GABAergic neurotransmission [8]. At later stages of development (>E8), however, network activity is driven by glutamate and GABA suggesting that there is a developmental switch in neurochemical transmission in the spinal cord [12].

Blockade of spinal cord activity with various pharmacological agents has been a valuable tool used to investigate the role of ongoing activity during normal development [3], [13]–[16]. For example, application of the GABA agonist muscimol can be used to prevent ongoing activity *in vivo* [13], [14]. However, concerns about drug specificity and side effects have often resulted in contradictory results [13], [14]. An alternative approach will be to silence spinal cord activity *in vivo* by using the avian replication-competent retroviral vector RCASBP (B) carrying the inward rectifier potassium channel Kir2.1. In many excitable cells, Kir2.1 expression plays a critical role in setting the resting membrane potential [17], [18]. Opening of Kir2.1 at resting potentials will decrease input resistance of the membrane and dampen electrical excitability, resulting in inhibition of neuronal activity in Kir2.1-expressing cells [4]. In this work we used the RCASBP(B) retroviral vector because it allows stable expression of a particular transgene in chicken embryos [19]–[21]. Stable expression of a transgene of interest occurs by infecting neuronal precursor cells in their mitotic stage, which will result in the insertion of the virus into the host genome followed by viral replication and further infection of other cells [22]. Our current results indicate that expression of the Kir2.1 transgene in the chicken spinal cord results in a significant inhibition of motor activity in chicken embryos. Inhibition of spinal cord activity is not accompanied by an increase in motoneuron loss and does not involve changes in cell capacitance or voltage-gated sodium channels. However, inhibition of ongoing activity in the spinal cord results in a significant decrease in the inactivation time constant of A-type potassium channels and a reduction of calcium-dependent potassium (K_Ca_) currents.

## Methods

### RCASBP (B) gene construct and virus production

Viral construction was carried out with the technical assistance of Dr. Sheryl White at the COBRE Molecular/Cellular core facility following a previously published protocol by Morgan and Fekete [23]. The human Kir2.1 sequence was obtained from an adenovirus vector kindly provided by Dr. E. Marban (Johns Hopkins University School of Medicine) [24]. GFP and Kir2.1 constructs were generated by cloning the genes of choice into the shuttle vector SLAX 12. Kir2.1 insertion was verified by sequence analysis. Viral stocks were generated by transfecting fibroblast cultures with the RCASBP(B) constructs and maintaining fibroblast cultures in modified L15 medium supplemented with 10% heat-inactivated horse serum, 50 U/ml penicillin, and 50 µg/ml streptomycin. Concentration of viral stocks was performed by ultracentrifugation at 90,000g at 4°C for 3 hr. After determining viral titers (>10^8^ infectious particles/mL), constructs were aliquoted and stored at −80°C until use.

### Viral Infections

Pathogen-free eggs were obtained from SPAFAS (Charles River Laboratories, Wilmington, MA) and incubated at 37°C. Embryos were staged according to Hamburger and Hamilton [25]. Prior to viral injections, a small window was cut in the shell directly above the embryo. Concentrated viral stocks were injected into the neural tube of E2 chicken embryos (corresponding to stage 8–10) using a fine tip pipette. In the chicken embryo, most lumbar motoneurons become postmitotic by E4 [26]. After injections, the window was closed with Scotch tape (3M, St. Paul, MN) and embryos were returned to the incubator. Embryos were incubated in a humidified incubator at 37°C until E8 (corresponding to stages 33–34) or E11 (corresponding to stage 37). The motility of surviving embryos was determined *in ovo* as the number of hindlimb kicks in a 3 min observation period [2]. No gross morphological differences were observed in control (non-injected) or infected embryos with RCASBP(B) or RCASBP(B)-Kir2.1 constructs.

### In ovo drug administration

The effect of tubocurare on motoneuron survival was assessed by daily drug application onto the vascularized chorio-allantoic membrane beginning at E5 as previously described [2], [13]. d-tubocurarine (2 mg/day), dissolved in sterile physiological saline containing (in mM): NaCl (139), KCl (3), MgCl_2_ (1), CaCl_2_ (3), NaHCO_3_ (17) was applied daily until E10 (corresponding to stage 36). This dose of D-tubocurarine has been reported to optimally inhibit spontaneous motility of the chicken hindlimb *in ovo* [2], [13].

### Islet immunohistochemistry and design-based stereology

Six segments of the lumbar enlargement (L1–L6) were removed at E10 and fixed in Zamboni's fixative [4% paraformaldehyde+15% picric acid in 0.1 M phosphate buffer saline (PBS)] at 4°C overnight, washed three times in PBS, and equilibrated in 30% sucrose/PBS overnight. Spinal cord tissue was embedded in OCT freezing medium, and 30 µm-cryostat sections were serially collected using a Leica cryostat. Sections were air dried for 5 min and postfixed in 4% paraformaldehyde for 30 min. Slides were washed three times in 0.1M PBS and blocked overnight in blocking solution (PBS containing 10% horse serum and 0.5% Triton X-100) at 4°C. Sections were incubated overnight at 4°C with *Islet* (1∶100 hybridoma supernatant, clone 39.405, Developmental Studies Hybridoma Bank, University of Iowa) diluted in blocking solution. This antibody recognizes expression of both *Islet* 1 and 2. Following three washes with PBS, sections were incubated with 0.5% hydrogen peroxide for 30 min to block endogenous peroxidase activity. After three more washes with PBS, slides were incubated for 2 hr at room temperature with a biotinylated goat anti-mouse antibody (1∶500, Vector Laboratories). Following three washes with PBS, slides were incubated with Vectastain ABC-HRP solution for 3–4 hr at room temperature. *Islet* staining was visualized by using a nickel/cobalt enhanced diaminobenzidine solution. After three washes, slides were mounted using AquaMount (Lerner Laboratories, Pittsburgh, PA). The number of *Islet*-positive neurons on both sides of the ventral spinal cord was counted in every fifth section using StereoInvestigator software (Microbrightfield Inc, Williston, VT). Images were obtained with a Nikon Eclipse E600W microscope coupled to a MicroFire video camera (Optronics) and with an x,y,z stage drive and position transducer (MAC 2000, Ludl Electronic Products, Ltd.). Under low magnification, the boundary of the motoneuron pool was identified and the boundary contour was drawn using the software-pointing device. A randomly generated sampling grid was placed over the contour area, containing 5–10 square counting frames (175×175 µm). Only *Islet*-stained nuclei within the counting frame and no contact with exclusion lines were counted using a 40× objective. The total number of motoneurons was obtained by adding together all counted neurons along L1–L6 spinal segments and multiplying by five.

### Hu and p27 gag immunocytochemistry

The lumbar spinal cord was isolated at E8, fixed in Zamboni's fixative overnight at 4°C, cryoprotected in 30% sucrose and embedded in OCT medium (Tissue-Tek) before freezing and cryostat sectioning into 14 µm-slices. Sections were blocked overnight in blocking solution at 4°C. Sections were then incubated overnight with various primary antibodies (mouse anti-Hu at 1∶250 or rabbit anti-p27 gag at 1∶2000) in blocking solution at 4°C. After three washes, sections were incubated for 1 h with the corresponding secondary antibodies (Alexa 488-conjugated anti-mouse and Cy3-conjugated anti-rabbit diluted at 1∶750, respectively). Sections were mounted in VectaShield medium (Vector Labs, Burlingame, CA) and visualized using a Nikon fluorescent microscope.

### PCR analysis

Total RNA was isolated with an RNA isolation kit according to the manufacturer's instructions (RNeasy Mini kit, Qiagen). To avoid amplification of genomic DNA, samples underwent DNAase treatment using the Qiagen RNase-free DNase kit. Isolated total RNA was used for cDNA synthesis by reverse transcription with an Omniscript reverse transcriptase kit (Qiagen). Amplification of Kir2.1 was performed with a SYBR green amplification kit (Applied Biosystems) using the following set of primers: forward primer (5′ TTTGGGAACGGGAAGAGTAAAGTC-3′), reverse primer (5′GAGGTACCGTTGCCCCTTCT-3′). PCR reactions were carried out as follows: one cycle of 95°C for 10 min followed by forty cycles of amplification (95°C/15 sec, 65.5°C/30 sec, and 70°C/30 sec). In each sample, Kir2.1 expression was normalized to β-actin. Quantification of β-actin cDNA was performed with a Taqman PCR master mix (Applied Biosystems) using the following set of primers and probe: forward primer (5′CACCTGAGCGCAAGTACTCTG -3′), reverse primer (5′ TCTGCTGGAAGGTGGACAG-3′), and TaqMan probe (5′TGGAGGCTCTATCCTGGCCTCCC-3′). PCR reactions consisted of one cycle of 50°C for 2 min, one cycle 95°C for 10 min, forty cycles of 95°C for 15 sec, and one cycle of 60°C for 1 min.

Quantification of choline acetyltransferase (ChAT) and glutamate decarboxylase (GAD 65) cDNA was performed by quantitative real-time PCR on an Applied Biosystems PRISM 7500 sequence detection system using specific primer pairs (Sigma Genosys) for each gene. For ChAT, we used the following set of primers: forward primer (5′ GTTGGTATGACAAACCCATG-3′), reverse primer (5′ TGAATCGGCTCGGAGT-3′); whereas for GAD detection we used the following primers: forward primer (5′ CAGCCTTGGGTATTGGTACAGA-3′), reverse primer (5′ CAGCTGTGGCACTCACTAGAA A-3′). Reactions were prepared for each cDNA using the Power SYBR Green PCR master mix according to the manufacture's instructions (Applied Biosystems). The cycling conditions were one cycle of denaturation at 95°C for 10 min, followed by forty cycles of amplification (95°C/15 sec, and 60°C/1 min). Each PCR reaction also included a reverse transcription negative control (without reverse transcriptase) to confirm the absence of genomic DNA and a non-template negative control. All PCR reactions were run simultaneously in duplicates. The identity of the PCR product was confirmed by sequencing at the Vermont Cancer Center. At the completion of the PCR reactions, the amount of target message in each sample was estimated from a threshold cycle number (C_T_), which is inversely correlated with the abundance of its initial mRNA. ChAT and GAD mRNA expression was normalized to β-actin to correct for differences in RNA concentration according to the delta-delta CT method [27].

### Synapse quantification

The number of synapses in the iliofibularis (IFIB) muscle was determined according to Dahm and Landmesser [28] by double labeling with Alexa 488-conjugated α-bungarotoxin to visualize postsynaptic AChR clusters and with SV2, an antibody that binds to presynaptic vesicle protein. Hindlimbs of chicken embryos were isolated at E8, fixed in Zamboni's fixative overnight at 4°C, incubated in 30% sucrose/PBS and embedded in tissue freezing medium (Richard Allen Scientific, Kalamazoo, MI) before cryostat sectioning. Serial 20 µm-transverse sections were collected on positively charged slides (Superfrost Plus, Fisher) and stored at −80°C before immunohistochemistry experiments were performed. Endogenous auto-fluorescence was blocked by incubating the sections with 50 mM NH_4_Cl/PBS for 1hr at room temperature. Cross sections were blocked in PBS with 0.5% Triton X-100 and 10% horse serum. After incubation in blocking solution for 1 hr at room temperature, sections were stained with Alexa 488-conjugated α-bungarotoxin (1∶500 dilution, Invitrogen) for 2 hr at room temperature. After several washes, sections were incubated with SV2 antibody (1∶100 dilution, Developmental Studies Hybridoma Bank, Iowa City, IA) overnight at 4°C. After three washes with PBS, slides were exposed to a Texas Red-conjugated secondary antibody (1∶750 dilution, Jackson ImmunoResearch, PA) for 3 hr at room temperature. Sections were visualized using a Deltavision Restoration Microscopy system equipped with an inverted Olympus IX70 microscope using a 40× oil objective. Digital images were captured using a Photometrics Coolsnap HQ camera. In order to quantify the number of synapses along the IFIB muscle, we counted the total number of co-localized AChR clusters with SV2 from every 5^th^ cross section. To determine whether errors were introduced due to changes in the size of the AChR clusters we also measured synaptic length. There were no significant changes in the length of AChR clusters in control embryos when compared with embryos infected with RCASBP(B) open vector or RCASBP(B)-Kir2.1 (7.2±0.8, 5.9±0.6, 5.2±0.6 µm, respectively).

### Motoneuron labeling, dissociation and cell culture

Labeling, dissociation and culture of chicken lumbar motoneurons were performed as previously described by Martin-Caraballo and Dryer [2]. Briefly, chicken lumbar motoneurons were retrogradely labeled *in ovo* with DiI (1 mg/ml in 20% ethanol and 80% saline). Dye injection into muscles of the thigh and foreleg was performed 24 hr before spinal cord dissociation. To enrich for motoneurons, only the ventral sections of the chicken spinal cord were excised into a Ca^2+^/Mg^2+^-free solution, mildly trypsinized (E8, 0.05%, 30 min; E11, 0.2% for 40 min), dissociated by trituration, and plated onto poly-d-lysine-coated glass coverslips. Basal culture medium consisted of Eagle's minimal essential medium (EMEM, BioWhittaker, Walkersville, MA) supplemented with 10% heat-inactivated horse serum, 2 mM glutamine, 50 U/ml penicillin and 50 µg/ml streptomycin. Cell cultures were used for electrophysiological recordings within 24 hr after seeding.

### Electrophysiology

LMNs were identified during patch clamp recordings using an inverted stage microscope equipped with epifluorescent optics and rhodamine filters. Motoneurons were identified by their large size and/or DiI labeling. Recordings were performed at room temperature (22–24°C). Recording electrodes were made from thin wall borosilicate glass (3–4 MΩ) and filled with a solution consisting of (in mM): 120 KCl, 2 MgCl_2_, 10 HEPES-KOH, and 10 EGTA, pH 7.4. Normal external saline for measurements of voltage-gated conductances contained (in mM): 145 NaCl, 5.4 KCl, 0.8 MgCl_2_, 5.4 CaCl_2_, 5 glucose, and 13 HEPES-NaOH, pH 7.4. The corresponding Ca^2+^-free solution was the same except that CaCl_2_ was replaced with an equimolar concentration of MgCl_2_. Voltage-activated Na^+^ currents were recorded in a Ca^2+^-free external solution. Na^+^ currents were evoked by applying a depolarizing voltage step to 0 mV from a holding potential of −80 mV. Whole cell recordings of K_Ca_ were performed as described previously [2]. Briefly, K_Ca_ currents were recorded by applying a 25 ms-depolarizing step to +30 mV from a holding potential of −40 mV in normal external saline and after 3 min incubation in Ca^2+^-free external saline. Net Ca^2+^-dependent outward currents were obtained by digital subtraction (control-Ca^2+^-free). We should note that recordings of Ca^2+^-dependent outward currents from a more depolarized holding potential (−40 mV) eliminates any possible contribution of A-type K^+^ channels to our recordings [2]. Inwardly rectifying K^+^ currents generated by Kir2.1 channel expression and A-type K^+^ currents were recorded in Ca^2+^-free external solution containing 600 nM TTX in order to block voltage-gated Ca^2+^ and Na^+^ channels, respectively. A-type K^+^ currents were isolated by applying a series of 200 ms-depolarizing steps to various potentials from either a holding potential of −100 or −40 mV. Net A-type K^+^ currents were obtained by digital subtraction of traces obtained from −100 and −40 mV holding potentials. Inwardly rectifying K^+^ currents were evoked by injecting an 850 ms-voltage ramp from −130 to +40 mV (slope 0.2 mV/ms). The composition of the external saline solution used to record voltage-activated Ca^2+^ currents was (in mM): tetraethylammonium chloride (145), CaCl_2_ (10), glucose (5), HEPES (10), pH 7.4 (with TEAOH). The pipette saline solution was (in mM): Cs-aspartate (140), MgCl_2_ (5), HEPES-CsOH (10), EGTA (10), MgATP (1), NaGTP (0.1), pH 7.4. Voltage-activated Ca^2+^ currents were generated by applying a 200 ms-voltage step to +30 mV from a holding potential of −40 mV. Voltage commands and data acquisition and analysis were performed with a MultiClamp 700B amplifier and Pclamp software (Axon Instruments, Foster City, CA). For quantitative analyses, we normalized for cell size by dividing current amplitudes by cell capacitance, determined by integration of the current transient evoked by a 10-mV depolarizing voltage step from a holding potential of −60 mV.

Current-clamp measurements were performed in the normal external saline solution. Resting membrane potential was determined by reading the Vm value immediately after switching to I = 0 in the patch-clamp amplifier. The passive (input resistance) and active membrane properties (action potential amplitude) of the motoneurons were recorded in the current-clamp configuration. Input resistance was determined from the voltage-deflection generated by injection of small hyperpolarizing currents [29]. Action potential amplitudes were measured from a holding potential of −60 mV following injection of a 1ms-depolarizing pulse. The ability of motoneurons to generate repetitive firing was investigated using longer depolarizing pulses (50 to 1000 ms).

### Intracellular free Ca^2+^ measurements

Changes in intracellular [Ca^2+^] resulting from activation of GABA receptors was detected with the ratiometric dye Fura-2 as previously described [30]. Briefly, cell cultures were incubated for 30 min with Fura-2 AM (5 µM, Molecular Probes, OR) and 0.2% pluronic acid in the dark. Cultures were washed and incubated for an additional 30 min in the dark to complete de-esterification of the dye. Cells were viewed with a Nikon microscope equipped with xenon epifluorescence optics and a 40× water immersion objective. Cells were illuminated with 340 and 380 nm light from a 75 W-xenon source and the emitted fluorescence was collected at 510 nm with a Hamamatsu CCD camera. Image collection and analysis were performed with the computer software Simple PCI (Compix Inc). Recorded Ca^2+^ signals were corrected for background fluorescence and presented as the ratio of the fluorescent peak signals generated at 340 and 380 (F_340_/F_380_). This ratio represents relative changes in intracellular [Ca^2+^] without conversion to absolute values of intracellular-free Ca^2+^. Drugs were applied for 30–60 sec with a ValveLink 8 perfusion system (AutoMate Scientific Inc, San Fransisco, CA). Recordings were performed in normal external saline solution without TTX. Control Ca^2+^ signals were generated by activation of voltage-gated Ca^2+^ channels with 30 mM extracellular K^+^.

### Data Analysis

Averaged data values are presented as mean±SEM. Where indicated, statistical analyses consisted of Student's unpaired *t*-test when single comparisons were made, or one-way ANOVA followed by *post hoc* analysis using Tukey's honest significant difference test for unequal *n* for comparisons between multiple age groups (Statistica software, Tulsa, OK). Throughout, *p*≤0.05 was regarded as significant. In every experiment, data were collected from a minimum of two platings (i.e. from multiple cultures).

### Chemicals and drugs

Poly-D-lysine, D-tubocurarine, tetrodotoxin, and trypsin were from Sigma (St. Louis, MO). Fura 2-AM and pluronic acid were purchased from Molecular Probes (Carlsbad, CA). Culture medium and supplements including serum were from BioWhittaker (Walkersville, MA).

## Results

**Figure 1:**
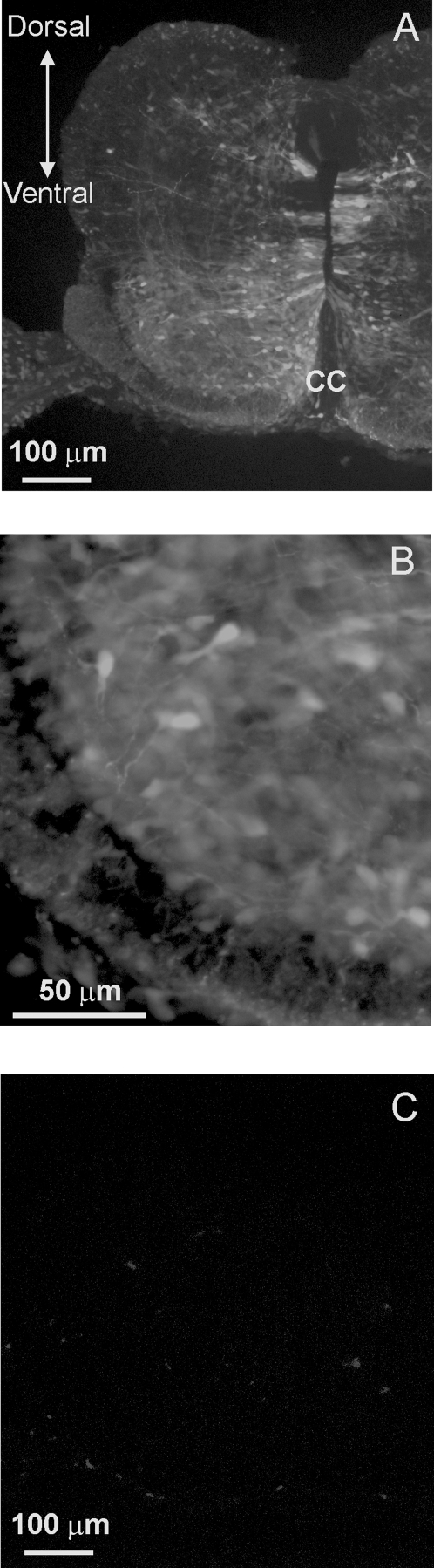
Expression of GFP transgene in the chicken spinal cord following retroviral infection with an RCASBP(B)-GFP construct. RCASBP(B)-GFP viral particles were injected into the developing neural tube at E2 (approximately 36 hr after incubation). *A)* Infected embryos with RCASBP(B)-GFP show strong fluorescent labeling throughout the whole spinal cord cross section. *B)* High magnification picture of the ventral spinal cord section shown in A. *C)* Embryos injected with RCASBP(B) open vector show no fluorescent signal. In these experiments, chicken embryos were infected with RCASBP(B)-GFP or RCASBP(B) open vector at E2. Embryos were allowed to develop until E8 (corresponding to stage 34) before tissue isolation and sectioning.

**Figure 2:**
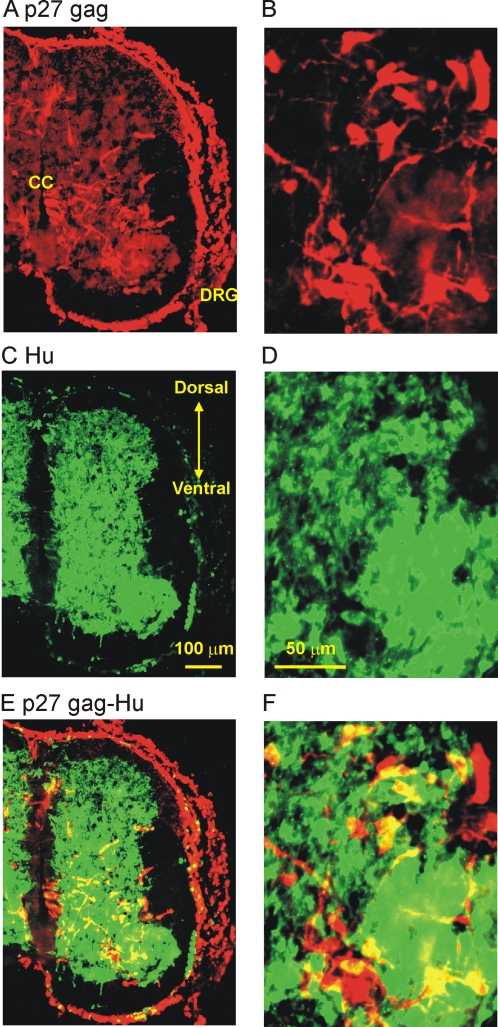
Immuno-labeling of chicken spinal cord for the viral gag p27 protein and the neuronal marker Hu. *A)* RCASBP(B)-Kir2.1 infected embryos show significant expression of viral p27 gag throughout the spinal cord. *C)* Hu staining is found mainly in the gray matter area of the spinal cord and in the DRG. *E)* Superimposed images from *A* and *C* showing double staining of spinal cord neurons with gag p27 viral protein and Hu. *B, D, F)* Higher magnification image of the ventral spinal cord for the sections represented in *A, C, E,* respectively. In these experiments, chicken embryos were infected with RCASBP(B)-Kir2.1 at E2. Embryos were allowed to develop until E8 before tissue isolation and sectioning.

**Figure 3:**
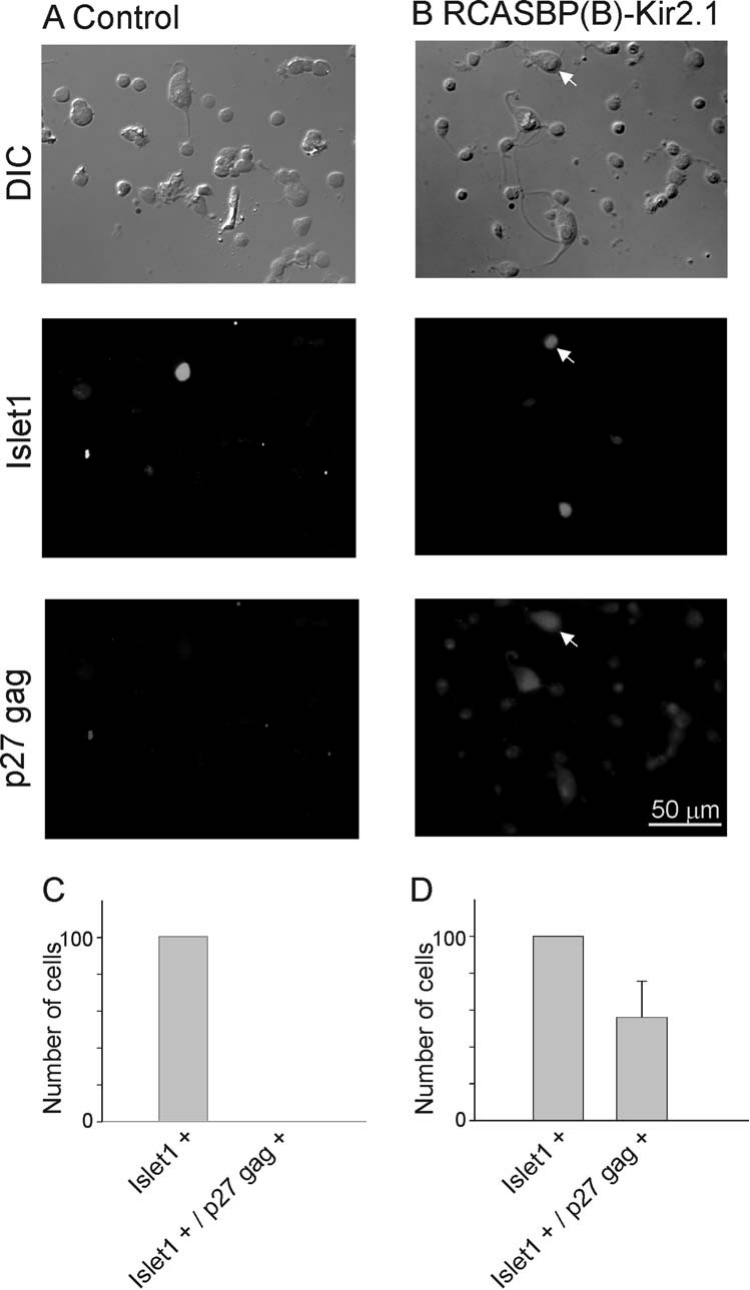
Immunolabeling of isolated ventral spinal cord neurons for p27 gag and Islet1/2. Ventral spinal cords from control (non-infected, *A*) and RCASBP(B)-Kir2.1 infected embryos (*B*) were isolated at E8. Dissociated neurons were plated on poly-D-lysine coated coverslips and inmunolabeled with the motoneuron marker *Islet*1/2 and the viral protein p27 gag. The arrow in *B* represents a typical motoneuron showing *Islet*1/2 and p27 gag labeling. *C &D)* Averaged number of double labeled neurons for *Islet*1/2 and p27 gag (as a percent of the total number of *Islet*-positive neurons) in control and RCASBP(B)-Kir2.1 infected embryos (n = 3).

Our initial experiments were designed to assess the extent of viral infection in the chicken spinal cord. An RCASBP(B) viral construct containing GFP was injected into the chicken neural tube at E2. At this stage motoneuron precursors are still dividing. Embryos were allowed to develop until E8 at which point the spinal cord was isolated to assess GFP expression. Infection of chicken embryos with the RCASBP(B)-GFP construct resulted in a significant expression of GFP throughout the whole spinal cord (Fig. 1). Cross sections of the lumbar spinal cord revealed extensive GFP fluorescence in the ventral spinal cord region (Fig 1A). GFP labeling appears to radiate from the ependymal layer surrounding the central canal. In the ventral horn of the spinal cord, many cells were GFP fluorescent (Fig. 1B). GFP fluorescence was also found in rostral areas of the spinal cord and outside of the central nervous system, including dorsal root ganglia, neural crest, and lens (not shown). No GFP fluorescent was detected in limb muscle tissue. None of the non-injected embryos showed GFP fluorescence (Fig. 1C). To investigate the extent of viral infection carrying the Kir2.1 transgene in neuronal cells, spinal cord sections from E8 embryos were double labeled for the viral gag protein p27 and the neuronal marker Hu (Fig. 2A–F). Viral gag p27 immunoreactivity was found throughout the spinal cord although it was not uniformly distributed (Fig. 2A & B). However, throughout the spinal cord, a significant number of Hu-positive cells became stained with p27 gag in the medial and ventral portions of the spinal cord (Fig. 2E & F). Uninfected embryos showed no labeling for the viral gag p27 protein (not shown). To quantify the number of infected motoneurons, ventral spinal cords were dissociated at E8 and isolated neurons were double stained for p27 gag and the motoneuron marker *Islet*1/2 (Fig. 3A & B). Only in RCASBP(B)-Kir2.1 infected embryos did we observe double-stained neurons for p27 gag and *Islet*1/2 (Fig. 3B). Approximately 55% of *Islet*1/2- positive neurons were also labeled with p27 gag, suggesting that approximately half of spinal motoneurons become infected with the viral construct (Fig. 3D). Non-injected embryos did not show any labeling for the viral gag p27 protein (Fig. 3A & C).

**Figure 4:**
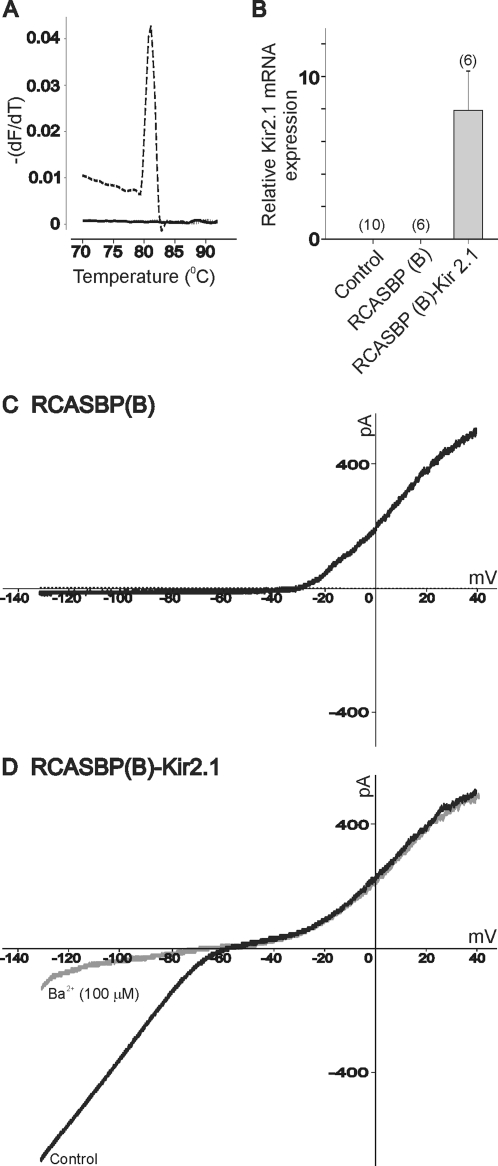
Kir2.1 expression in the chicken spinal cord. *A)* Typical melting curve from samples obtained from an RCASBP(B)-infected embryo (continuous line) or an RCASBP(B)-Kir2.1-infected embryo (discontinuous line). Fluorescence melting peaks were obtained by plotting the negative derivative of the fluorescence signal over temperature (-dF/dT) as a function of temperature (T). Notice the sharp peak in the sample obtained from an RCASBP(B)-Kir2.1- but not RCASBP(B)-infected embryo, indicating the presence of one PCR product. *B)* Quantification of Kir2.1 mRNA expression by real time PCR in chicken ventral spinal cords isolated at E8. *C & D)* Whole cell recordings from RCASBP(B) and RCASBP(B)-Kir2.1 infected embryos. Currents were evoked by an 850 ms-voltage ramp from −130 mV to +40 mV. Notice that incubation with 100 µM barium ions causes a significant reduction in the inwardly rectifying current (in D). Chicken embryos were infected with RCASBP(B)-Kir2.1 or RCASBP(B) open vector at E2. Controls consisted of non-injected embryos. Motoneurons were acutely isolated at E8.

Does infection of chicken spinal cord with the RCASBP(B)-Kir2.1 construct result in increased Kir2.1 expression? Expression of Kir2.1 in chicken embryos was quantified by real time PCR using E8 ventral spinal cords. As shown in Fig. 4A (discontinuous line) from a representative sample of a chick embryo infected with RCASBP(B)-Kir2.1, the melting curve show a single peak, indicating the presence of a single PCR product. No amplification product was detected in samples obtained from RCASBP(B)-infected embryos (Fig. 4A, continuous line) or non-infected controls (not shown). Quantification of Kir2.1 mRNA expression by real time PCR shows no detectable levels of Kir2.1 mRNA in non-injected controls and embryos injected with the RCASBP(B) open vector (Fig. 4B). Kir2.1 mRNA expression was only detected in embryos infected with the RCASBP(B)-Kir2.1 construct (Fig. 4B). Little expression of Kir2.1 mRNA seems to confirm data from our electrophysiological recordings that show no inward rectification in acutely isolated lumbar motoneurons from non-injected embryos or embryos injected with the RCASBP(B) open vector (Fig, 4C). Functional expression of Kir2.1 channels was assessed in isolated lumbar motoneurons by recording whole cell currents generated by injection of an 850 ms-voltage ramp from −130 to +20 mV (Fig. 4C & D). In chicken embryos injected with RCASBP(B) open vector, injection of hyperpolarizing voltage steps or a voltage ramp did not show any inward current at hyperpolarizing voltage potentials between −130 and −60 mV (total of 28 neurons recorded from 4 different embryos, Fig. 4C). Consistent with an increased expression of Kir2.1 mRNA, whole cell recordings revealed the presence of an inward rectifying current in motoneurons from RCASBP(B)-Kir2.1 injected embryos (n = 12 out of 28 neurons recorded, Fig. 4D). Kir2.1 positive motoneurons were defined as those having an inward current above 50 pA at −110 mV and a non flat IV between −130 and −40 mV. The inwardly rectifying current reverses in polarity at −70.3±6.2 mV (n = 7), close to the potassium reversal potential calculated according to the Nernst equation (−80.8 mV) under our specific recording conditions. Consistent with the high sensitivity of Kir2.1 channels to low concentrations of barium ions [31], application of 100 µM barium blocked the inwardly rectifying current by ∼80% (76.8±15.2% at −110 mV, n = 4; Fig. 4D).

**Figure 5:**
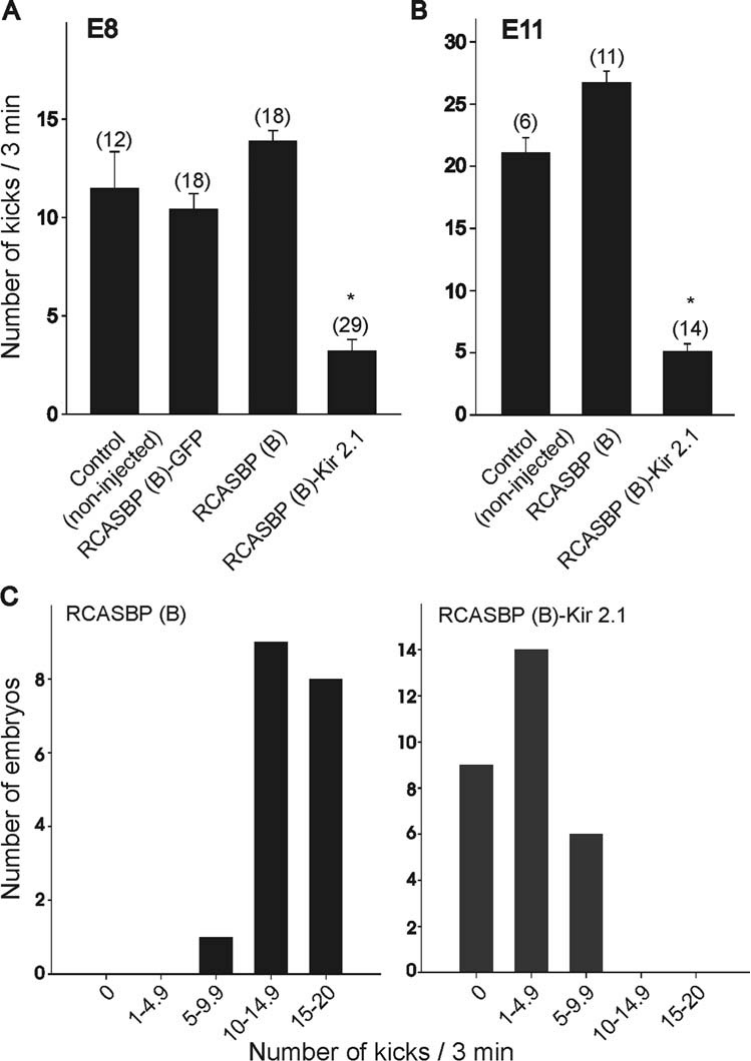
Effect of Kir2.1 expression on embryonic movement at E8 and E11 in chicken embryos infected with RCASBP(B), RCASBP(B)-GFP, or RCASBP(B)-Kir2.1. Controls consisted of non-injected embryos. *A & B)* Average number of kicks in a 3 min observation period in E8 (A) and E11 (B) chicken embryos injected with different RCASBP(B) constructs. The number of embryos analyzed under different condition is given above each bar. *C)* Plot showing the distribution of the number of kicks in RCASBP(B) and RCASBP(B)-Kir2.1 infected embryos at E8.

To assess the effect of Kir2.1 transgene on motor activity, we counted the number of spontaneous kicks generated during a 3 min interval in chick embryos isolated at E8 or E11. In chicken embryos, limb movements or kicks are driven by spontaneous network activity in the spinal cord [39]–[40]. At E8, the number of kicks in chicken embryos infected with the RCASBP(B) open vector or RCASBP(B)-GFP constructs was not significantly different from the number of kicks observed in non-injected controls (control = 11.5±1.9, GFP = 10.4±0.8, RCASBP(B) = 13.9±0.5, Fig. 5A). However, expression of the Kir2.1 transgene caused a significant reduction in the number of spontaneous movements (RCASBP(B) = 13.9±0.5, RCASBP(B)- Kir2.1 = 3.2±0.6, Fig. 5A). Kir2.1 expression resulted in a leftward shift in the distribution of number of kicks when compared with embryos injected with the open vector (Fig. 5C). Expression of Kir2.1 was also able to downregulate spontaneous motor activity in older embryos (E11). As represented in figure 5B, infections of chicken embryos with RCASBP(B)-Kir2.1 resulted in a significant reduction in the number of kicks at E11 (RCASBP(B) = 26.7±1.5, RCASBP(B)-Kir2.1 = 5.0±0.6).

**Figure 6:**
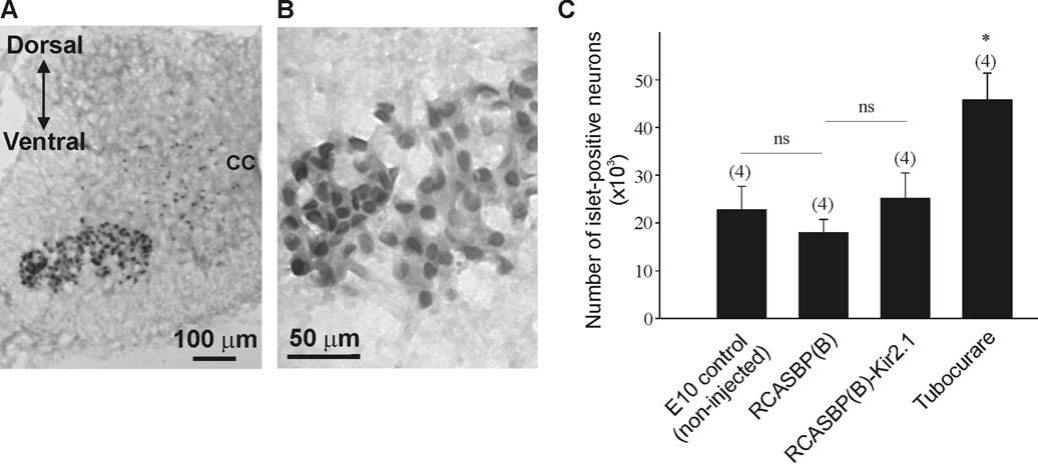
Effect of Kir2.1 expression on neuronal survival in non-injected, and RCASBP(B) and RCASBP(B)-Kir2.1 infected embryos. *A)* *Islet* staining in the lumbar spinal cord of a Kir2.1-infected embryo. *B)* Higher magnification image of the ventral spinal cord cross section represented in *A.* ** *C)* Inhibition of electrical activity in RCASBP(B)- Kir2.1 infected embryos does not alter motoneuron survival, whereas application of the neuromuscular blocker tubocurare caused a significant increase in the number of surviving neurons. The number of *Islet*-positive neurons on both sides of the lumbar spinal cord was counted using design-based stereology. Chicken embryos were infected with RCASBP(B)-Kir2.1 or RCASBP(B) open vector at E2 and motoneuron survival was assessed at E10 from six lumbar segments (L1–L6).

Are the changes in chicken motility the result of motoneuron loss? To investigate whether infection of chicken embryos with an RCASBP(B) construct containing a Kir2.1 transgene may adversely affect motoneuron survival, we counted the number of *Islet*-positive neurons in the ventral spinal cord using design-based stereology (Fig. 6A, B). *Islet* is a marker of postmitotic spinal motoneurons [32]. Cell count of *Islet*-positive neurons was performed at E10 when programmed cell death is completed in the chicken spinal cord [33]. Cell counts of *Islet*-positive neurons in RCASBP(B)-infected embryos did not result in a significant change in the number of surviving motoneurons compared with non-injected embryos, suggesting that viral expression does not have any toxic affect on developing motoneurons (E10 control = 22752±2446, RCASBP(B) = 17907±1377, Fig. 6C). In RCASBP(B)-Kir2.1 infected chicken embryos, blockade of ongoing activity does not result in a statistically significant change in the number of *Islet*-positive neurons compared with RCASBP(B)-infected embryos (RCASBP(B) = 17907±1377 vs. RCASBP(B)-Kir2.1 = 25107±2671, Fig. 6C). Previous studies have shown that chronic treatment of chicken embryos with the nicotinic receptor antagonist d-tubocurare stimulates motoneuron survival by increasing intramuscular branching of lumbar motoneurons and thereby potentially increased access to target-derived trophic factors [34]–[35]. Consistent with this evidence, we also observed a significant increase in the number of *Islet*- positive neurons following chronic treatment with tubocurare (E10 control = 22752±2446 vs. Tubocurare = 45772±2834, Fig. 6C).

**Figure 7:**
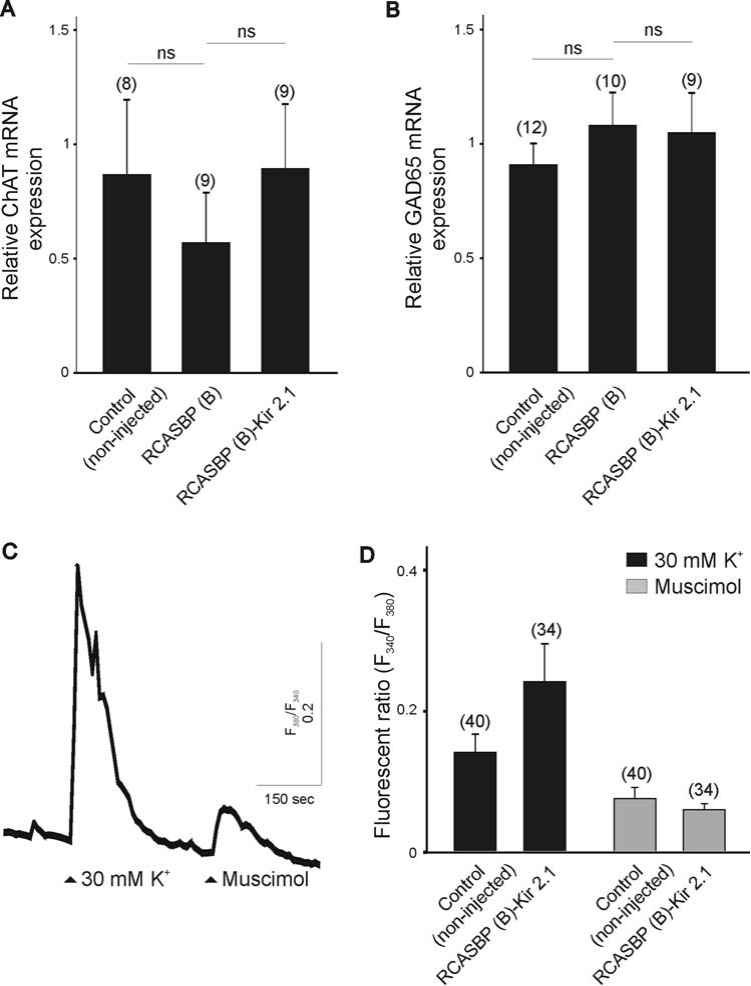
Effect of Kir2.1 expression on neurotransmitter specification and function in chicken spinal cord neurons. Inhibition of spinal cord activity does not alter the expression of the acetylcholine and GABA synthesizing enzyme ChAT (choline acetyltransferase, *A*) and GAD (glutamate decarboxylase, *B*) mRNA at E8. *C)* Stimulation of chicken spinal motoneurons with the GABA agonist causes a significant increase in intracellular Ca^2+^. *D)* There was no significant change in the muscimol evoked- Ca^2+^ signals measured in control or RCASBP(B)-Kir2.1 infected embryos at E8. Acutely isolated motoneurons were incubated with the ratiometric Ca^2+^ indicator Fura-2 for 30 min and stimulated with 100 µM muscimol for 30 sec. Stimulation with 30 mM K^+^ served as a control.

To investigate whether inhibition of network activity in the chicken spinal cord can alter neurotransmitter expression, we studied the expression of the acetylcholine and GABA synthesizing enzymes choline acetyltransferase (ChAT) and glutamate decarboxylase (GAD), respectively. Previously, it has been reported that cholinergic and GABAergic neurotransmission mediate early network activity in the chicken spinal cord (between E4–E6), however, activation of both glutamate and GABA receptors is critical for the generation of spontaneous activity by E10 [8], [12]. To determine whether inhibition of electrical activity in chicken embryos has any effect on neurotransmitter expression at early stages of spinal cord development we determined the relative expression of ChAT and GAD mRNA by real time PCR in chicken ventral spinal cords isolated at E8 (Fig. 7A, B). No significant differences in the relative abundance of ChAT and GAD mRNA were found between non-injected or RCASBP(B)-infected embryos (Fig. 7A & B). No statistically significant differences in ChAT and GAD expression were also detected between RCASBP(B) and RCASBP(B)-Kir2.1 infected embryos (Fig. 7A & B) suggesting that reduction in activity does not alter neurotransmitter expression between E8 and E11. Although GABA and glycine are the main inhibitory neurotransmitters in the mature spinal cord, at early stages of development, these neurotransmitters mediate membrane depolarization and excitation due to an immature chloride gradient [36]. The depolarizing effect of GABA can be indirectly assessed by measuring changes in intracellular Ca^2+^. In isolated chicken spinal cord motoneurons, application of the GABA agonist muscimol causes a small but nonetheless noticeable increase in intracellular Ca^2+^ (Fig. 7C). To investigate whether inhibition of spinal cord activity could alter the depolarizing effect of GABA receptor activation, we compared the effect of muscimol in isolated motoneurons from control and RCASBP(B)-infected embryos (Fig. 7D). There was no significant change in the fluorescence ratios of motoneurons of control and RCASBP(B)-Kir2.1 infected embryos after stimulation with 100 µM muscimol (RCASBP(B) = 0.20±0.04, RCASBP(B)-Kir2.1 = 0.22±0.09, Fig. 7D) or 30 mM high K^+^ solution (RCASBP(B) = 0.92±0.24, RCASBP(B)-Kir2.1 = 1.24±0.21, Fig. 7D).

**Figure 8:**
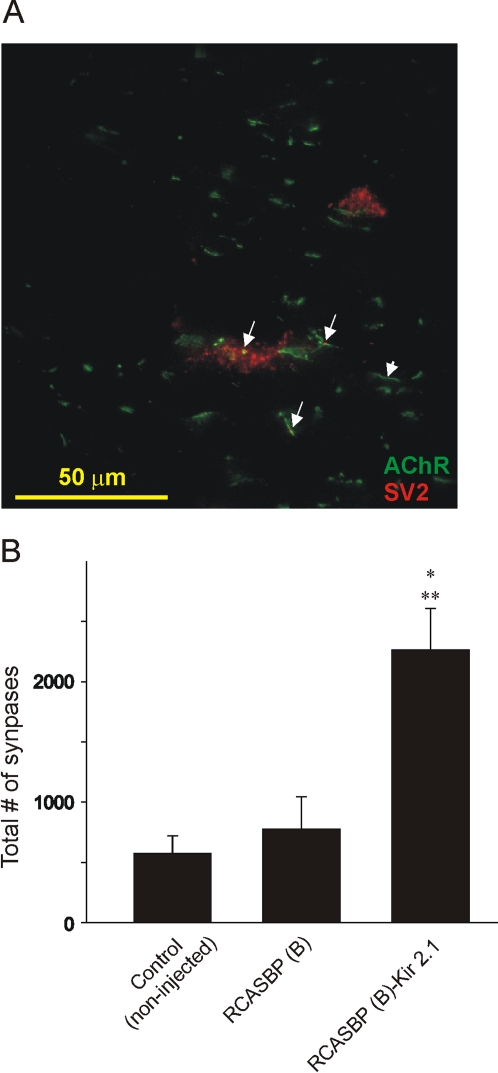
*A)* Distribution of AChR clusters and presynaptic terminals in a cross section of the iliofibularis (IFIB) muscle as revealed by double labeling with Alexa 488- conjugated α-bungarotoxin and SV2. Large arrows indicate co-localization of AChR clusters and presynaptic terminals, whereas the short arrow indicates an AChR clusters alone. *B)* The total number of synapses (as determined by co-localization of AChR clusters and SV2-labeling) was determined in control (non-injected), or chicken embryos infected with RCASBP(B) open vector or RCASBP(B)-Kir2.1. Chicken embryos were isolated at E7. Notice there are no significant differences in the number of synapses between non injected and RCASBP(B)-infected embryos. Infection of chicken embryos with RCASBP(B)-Kir2.1 results in a significant increase in the number of synapses along the IFIB muscle at E7. * denotes *p*≤0.05 vs. control (non-injected); ** denotes *p*≤0.05 vs. RCASBP(B)-infected embryos (n = 3).

To determine whether inhibition of spontaneous motor activity in chicken embryos infected with RCASBP(B)-Kir2.1 disrupt synaptic connectivity between motor nerve terminals and muscle fibers we measured the number of synapses in the iliofibularis (IFIB) muscle of the hindlimb. Synapses were defined as structures where postsynaptic nicotinic acetylcholine receptor (AChR) clusters labeled with Alexa 488-conjugated α-bungarotoxin were co-localized with presynaptic terminal vesicles stained with an SV2 antibody (Fig. 8A). The total number of synapses along the IFIB muscle was determined in non-injected embryos and embryos infected with RCASBP(B) open vector or RCASBP(B)-Kir2.1 constructs (Fig. 8B). To determine the number of co-localized AChR clusters and presynaptic terminals, embryos were isolated at E7 (corresponding to stage 32) and the number of synapses was determined during the linear phase of synapse formation in hindlimb muscles before it reaches a maximum at E8 (Dahm & Landmesser, 1991). Results from a total of 3 embryos in each group indicates that the number of synapses in control and embryos infected with RCASBP(B) was not significantly different (control = 577±144, RCASBP(B) = 778±269, Fig. 8B). However, there was a 3-fold increase in the number of synapses in embryos infected with RCASBP(B)-Kir2.1 (RCASBP(B)-Kir2.1 = 2264±345, Fig. 8B) when compared with control or RCASBP(B) open vector-infected embryos.

**Figure 9:**
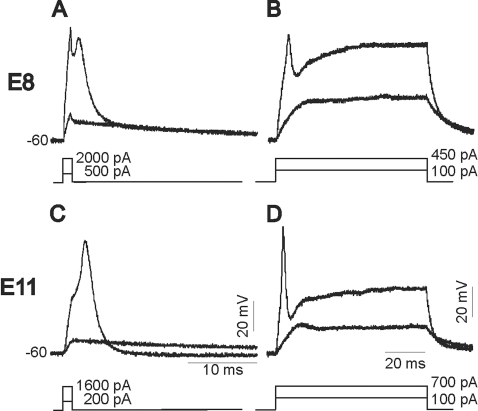
Typical action potentials generated in E8 and E11 chicken spinal motoneurons infected with RCASBP(B). Motoneurons were obtained from chicken embryos isolated at E8 or E11. Individual action potentials were generated by injection of 1 ms-depolarizing currents (*A* & *C*). Injection of long depolarizing current pulses (50 ms, *B* & *D*) only generated one action potential. Bottom trace in *A*–*D* represents the stimulation protocol used and the amount of current injected.

Does Kir2.1 expression alter the electrical properties of chicken spinal motoneurons? In order to record the electrical excitability of spinal motoneurons we recorded membrane responses to current injection in the current-clamp configuration. Motoneurons were acutely isolated at E8 or E11 and the membrane responses of RCASBP(B)-infected embryos were compared with those found in RCASBP(B)-Kir2.1. At all ages tested, infection of chicken embryos with RCASBP(B)-Kir2.1 resulted in more negative resting membrane potentials (Table 1). Kir2.1 expression also resulted in a significant reduction in input resistance in E11 motoneurons (Table 1). Single action potentials were generated by injection of brief depolarizing pulses (1 ms). Injection of brief depolarizing currents resulted in the generation of action potentials in motoneurons isolated at E8 or E11 (Fig. 9A & C). Action potential amplitudes in E8 motoneurons were significantly lower than at E11 (Table 1). There were no significant differences in the action potential amplitude at any age between RCASBP(B) and RCASBP(B)-Kir2-infected motoneurons (Table 1). Injection of longer depolarizing pulses [50 ms (Fig. 9B & D) or 1000 ms (not shown)] resulted in the generation of a single action potential. Therefore, we were not able to study the effect of Kir2.1 expression on repetitive firing.

**Table 1:** Changes in resting membrane potential, input resistance and action potential amplitude in E8 and E11 motoneurons isolated from chicken embryos infected with RCASBP(B) or RCASBP(B)-Kir2.1.

	E8		E11	
	RCASBP(B)	RCASBP(B)-Kir2.1	RCASBP(B)	RCASBP(B)-Kir2.1
Resting membrane potential (mV)	−55.8±3.4 (n = 13)	−64.3±1.5* (n = 12)	−53.1±2.6 (n = 15)	−66.4±2.4** (n = 9)
Input resistance (MOhm)	1362±360 (n = 9)	1486±231 (n = 11)	1951.2±197.0 (n = 13)	1224.3±251.9** (n = 9)
Action potential amplitude (mV)	61.0±4.0 (n = 13)	59.8±3.4 (n = 12)	71.9±3.4* (n = 12)	72.2±4.7# (n = 9)

* , p<0.05 vs. E8 RCASBP(B);

** , p<0.05 vs. E11 RCASBP(B);

# , p<0.05 vs. E8 RCASBP(B)-Kir2.1

**Figure 10:**
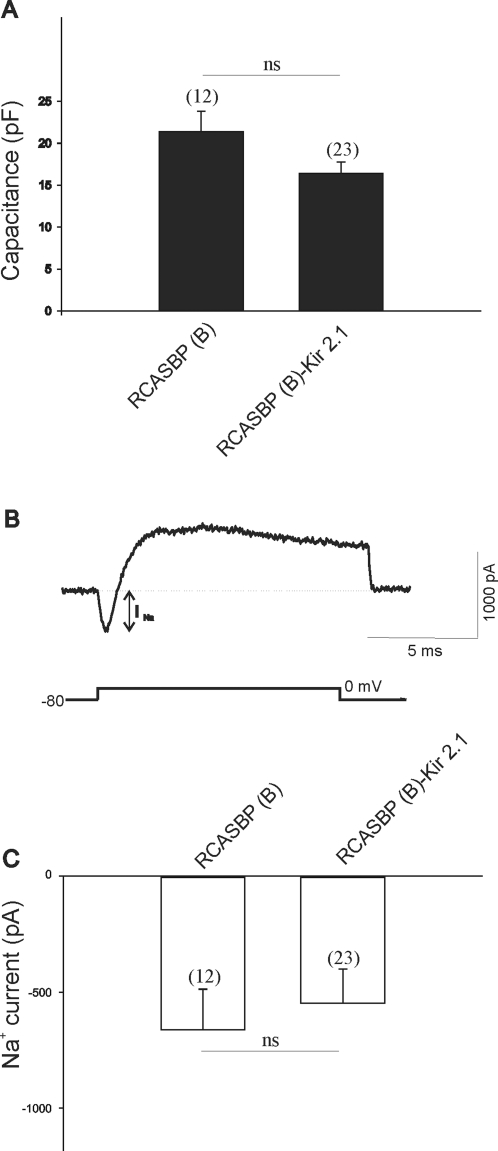
Effect of Kir2.1 expression on cell capacitance and sodium current amplitude in RCASBP(B) and RCASBP(B)-Kir2.1 infected embryos. *A)* Cell capacitance is not affected by inhibition of spinal cord activity in RCASBP(B)-Kir2.1 infected embryos. *B)* Current generated by a 25 ms-depolarizing step to 0 mV from a holding potential of −80 mV (stimulation protocol is shown as bottom trace). Note the presence of a fast inward current (or sodium current, I_Na_) that precedes opening of potassium channels and the generation of an outward potassium current. *C)* No changes in sodium current amplitude were detected in RCASBP(B) and RCASBP(B)-Kir2.1 infected embryos. Recordings were performed in acutely isolated motoneurons at E8.

**Figure 11:**
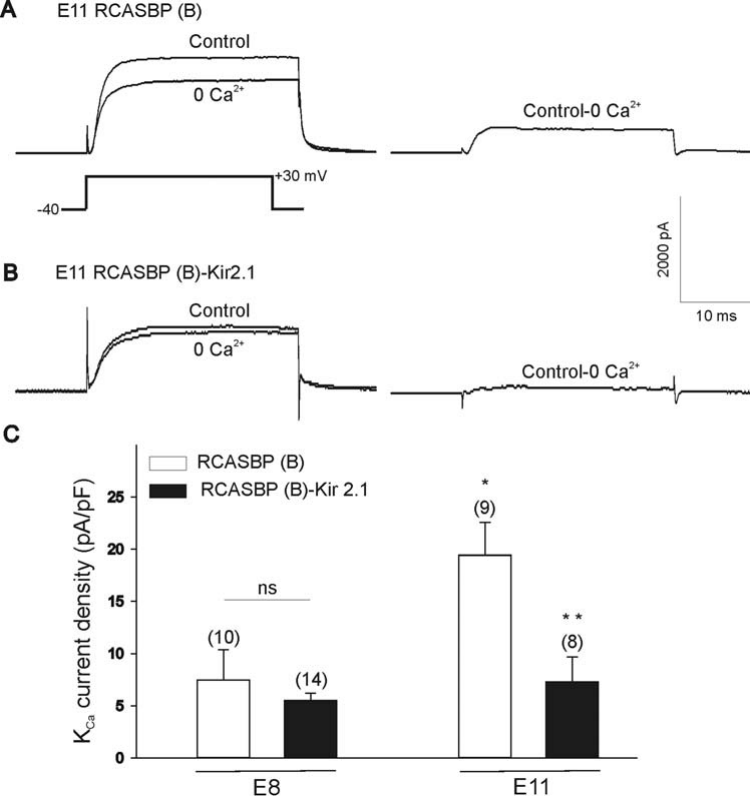
Effect of Kir2.1 expression on the functional expression of K_Ca_ currents in embryonic lumbar motoneurons developing in vivo. *A, B)* Outward currents in E11 motoneurons from chicken embryos infected with RCASBP(B) or RCASBP(B)-Kir2.1 constructs, respectively. Outward currents were evoked in control and Ca^2+^-free saline (right traces in *A* and *B*) following a 25 ms depolarizing step to +30 mV from a holding potential of −40 mV (stimulation protocol is shown as the left bottom trace in *A*). Net Ca^2+^-dependent outward currents were obtained by digital subtraction of raw traces (right traces in *A* and B). *C)* Kir2.1 expression resulted in a significant inhibition of K_Ca_ current density in E11 lumbar motoneurons. Notice that at K_Ca_ expression is significantly low in chicken motoneurons isolated at E8 in both RCASBP(B) or RCASBP(B)-Kir2.1 infected embryos. K_Ca_ expression increased ∼3 fold in RCASBP(B)-infected embryos by E11 but was significantly reduced in RCASBP(B)-Kir2.1 infected embryos. * represents p≤0.05 vs. E8 RCASBP(B), ** p≤0.05 vs. E11 RCASBP(B)-Kir2.1.

We have previously demonstrated that pharmacological inhibition of ongoing electrical activity in the chicken spinal cord plays a significant role in regulating electrical differentiation of developing LMNs [2], [37]. To determine whether decreased electrical activity in chicken embryos results in a significant change in the electrical properties of LMNs we conducted whole cell recordings of acutely isolated cells. Motoneurons were isolated at E8 or E11 from embryos infected with the RCASBP(B) open vector or RCASBP(B)-Kir2.1 constructs. Infection of chicken embryos with RCASBP(B)-Kir2.1 did not result in a significant change in cell size as revealed by measurements of cell capacitance in E8 motoneurons (cap RCASBP(B) = 21.4±2.5 pF, cap RCASBP(B)-Kir2.1 = 16.4±1.3 pF, Fig. 10A). Cell capacitance in RCASBP(B) or RCASBP(B)-Kir2.1-injected embryos was similar to that previously reported for non-injected spinal cord neurons [2]. In the chicken spinal cord, Na^+^ currents mediate action potential spikes at E6 [38]. To determine whether paralysis of spinal cord activity with Kir2.1 has any effect on Na^+^ conductances we measured the current generated by injection of depolarizing voltage step to 0 mV from a holding potential of −80 mV. Expression of Kir2.1 transgene did not alter the amplitude of voltage-gated sodium currents in acutely isolated motoneurons (I_Na_ RCASBP(B) = −663±176 pA, I_Na_ RCASBP(B)-Kir2.1 = −547±147 pA, Fig. 10 B & C). Expression of Kir2.1 transgene, however, did have a noticeable effect on two types of potassium conductances. Previous findings indicate that expression of Ca^2+^-dependent K^+^ (K_Ca_) channels is developmentally regulated in chicken spinal motoneurons [2]. Thus, the functional expression of K_Ca_ channels increases ∼3 fold between E8 and E11 (Fig. 11C). Similar to our previous findings in non-infected chicken embryos, in spinal cords transfected with RCASBP(B) open vector we also observed a near 3 fold increase in K_Ca_ channel density (I_KCa_ E8 RCASBP(B) = 7.5±2.9 pA/pF, I_KCa_ E11 RCASBP(B) = 19.4±3.1 pA/pF, Fig. 11A, B, C). The age-dependent increase in K_Ca_ expression was reversed in chicken embryos infected with RCASBP(B)-Kir2.1 suggesting that inhibition of electrical activity prevents normal development of K_Ca_ channels (I_KCa_ E8 RCASBP(B)-Kir2.1 = 5.5±0.7 pA/pF, I_KCa_ E11 RCASBP(B)-Kir2.1 = 7.3±2.4 pA/pF, Fig. 11A, B, C). K_Ca_ channel activation required Ca^2+^ influx via voltage-activated Ca^2+^ currents. Therefore, inhibition of K_Ca_ expression could be attributed to activity-evoked changes in voltage-activated Ca^2+^ currents. To investigate this possibility, we recorded voltage-activated Ca^2+^ currents in E11 motoneurons isolated from embryos infected with RCASBP(B) or RCASBP(B)-Kir2.1. Under our recording conditions (see Methods), Ca^2+^ current densities did not change significantly between RCASBP(B) and RCASBP(B)-Kir2.1-infected neurons. In RCASBP(B)-infected embryos, Ca^2+^ current density was −10.6±2.6 pA/pF (n = 10), whereas in RCASBP(B)-Kir2.1 infected embryos Ca^2+^ current density was −8.7±2.8 pA/pF (n = 9, p>0.05). These results suggest that changes in K_Ca_ expression are not due to changes in Ca^2+^ influx via voltage-activated Ca^2+^ channels.

**Figure 12:**
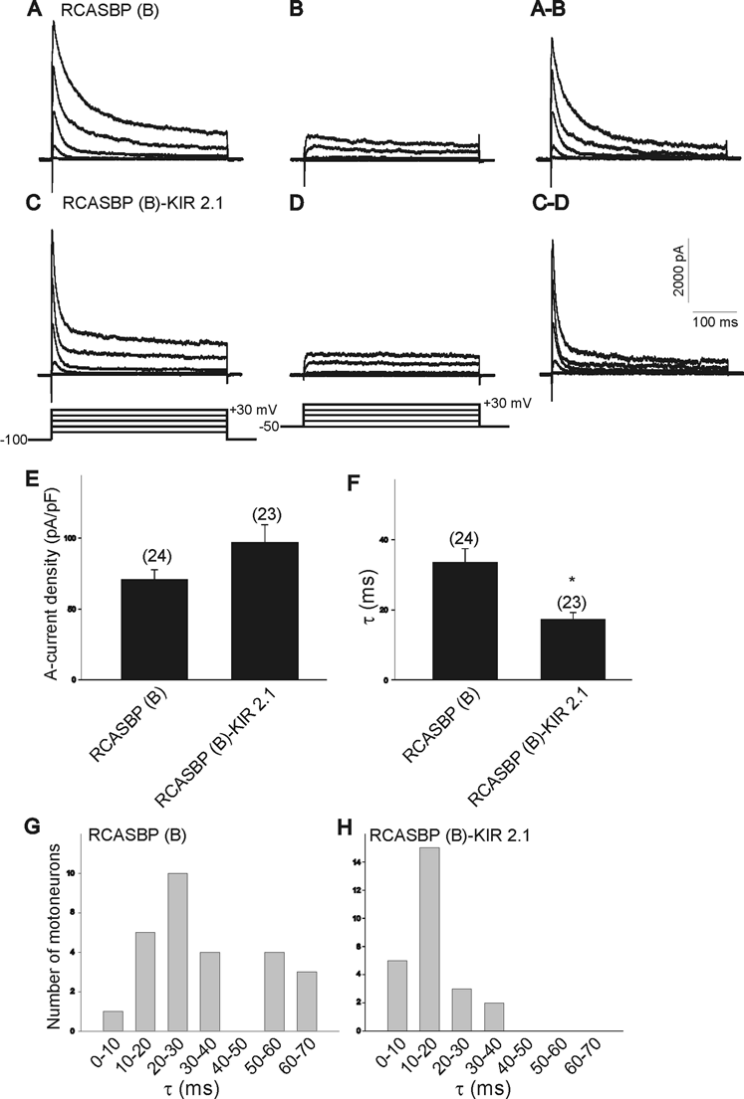
Effect of Kir2.1 expression on the kinetics of A-type K^+^ channels. *A–D)* Outward K^+^ currents generated in E8 lumbar motoneurons from chicken embryos injected with the RCASBP(B) and RCASBP(B)-Kir2.1 constructs. Outward K^+^ currents were evoked following a series of 10 mV-depolarizing voltage steps from a holding potential of −100 mV (*A* and *C*) or from a holding potential of −40 mV (*B* and *D*). Stimulation protocol used in each case is shown as bottom traces in *C* and *D*. Net A-type K^+^ currents were obtained by digital subtraction of traces obtained from −100 and −40 mV holding potentials (*A–B* and *C–D*). *E)* Blockade of spinal cord activity has no significant effect on A-type K^+^ current density (*p* = 0.08). *F)* Disruption of spinal cord activity results in a significant reduction in the inactivation time constant of A-type K^+^ currents (τ, *p* = 0.04 vs. RCASBP(B)). *G–H)* Histograms of inactivation time constants in acutely isolated motoneurons from chicken embryos infected with the RCASBP(B) or RCASBP(B)-Kir2.1 constructs. Changes in A-type K^+^ current density and inactivation time constant were calculated from whole-cell currents evoked by a step pulse to +10 mV.

Blockage of ongoing activity in the spinal cord also resulted in a significant change in the kinetics of A-type K^+^ currents (Fig. 12). The A-type K^+^ current is a transient outward current with fast activation and inactivation kinetics (Fig. 12A–D). Chicken LMNs already express A-type K^+^ channels by E4 and there is a developmental increase in the functional expression of these channels between E4 and E11 [37]–[38]. Our present results show the presence of a significant A-type current component in RCASBP(B) or RCASBP(B)-Kir2.1-injected embryos (Fig. 12 A–B and C–D, respectively). Blockade of spinal cord activity with Kir2.1 did not have any effect on A-type current density (I_A_ RCASBP(B) = 70.7±7.2 pA/pF, I_A_ RCASBP(B)-Kir2.1 = 96.7±12.5 pA/pF, Fig. 12E). Infection of chicken embryos with RCASBP(B)-Kir2.1 did not result in any significant change in the A-current activation time when compared with RCASBP(B)-infected embryos (RCASBP(B) = 2.33±0.11 ms, n = 18 vs. RCASBP(B)-Kir2.1 = 2.29±0.07, n = 18, p>0,05). However, paralysis of spinal cord activity resulted in a significant increase in the inactivation rate of the transient A-current component, suggesting that blockade of electrical activity generates A-type K^+^ channels with faster inactivation kinetics (τ RCASBP(B) = 33.6±3.9 ms, τ RCASBP(B)-Kir2.1 = 17.4±1.9 ms, Fig. 12F). Blockade of spinal cord activity resulted in a rightward shift in the histogram of inactivation time constants of RCASBP(B)-Kir2.1-infected embryos when compared with embryos infected with RCASBP(B) (Fig. 12G and H).

## Discussion

In this study we have examined the effect of chronic inhibition of spinal cord activity on motoneuron survival and electrical differentiation. Our present results indicate that infection of chicken embryos with an RCASBP(B)-Kir2.1 vector causes a significant upregulation of Kir2.1 expression in the chicken spinal cord, which ultimately prevents the generation of spontaneous motor activity in the developing chicken embryo. This effect is not caused by changes in motoneuron survival or neurotransmitter expression. However, inhibition of ongoing activity in the spinal cord results in changes in the electrical properties of the motoneurons. Thus, downregulation of spinal cord activity prevents normal expression of functional Ca^2+^-dependent K^+^ channels and alters the decay time constant of A-type K^+^ currents.

Contrary to electroporation and other transfection techniques, retroviral vectors are a useful tool for the introduction and long-lasting expression of foreign genes in the developing chicken embryo [19]–[21]. The present results indicate that infection of chicken embryos with an RCASBP(B)-Kir2.1 viral construct results in transgene expression throughout the spinal cord. Infection of chicken embryos was carried out at E2, when motoneurons precursors are undergoing cell division, which is required in order for the viral DNA to become integrated in the host genome. Embryos exposed to RCASBP(B)-Kir2.1 express significant levels of functional Kir2.1 channels which leads to significant reduction in motor activity in developing chicken embryos until E11, the last stage of development studied. An obvious advantage of this approach is also the lack of toxicity. Our present results indicate that infection of chicken spinal cords with the RCASBP(B)-Kir2.1 did not alter the number of *Islet*-positive neurons, suggesting that viral infection and expression of Kir2.1 channels does not have an adverse effect on motoneuron survival. Infection of chicken spinal neurons with a RCABP(B)-Kir2.1 construct, however, induced a significant reduction in the number spontaneous kicks generated *in ovo*. These results suggest that infection of chicken embryos with RCABP(B)-Kir2.1 can be a valuable tool to block electrical activity using a genetic rather than a pharmacological approach.

Expression of Kir2.1 channels causes a significant downregulation of spontaneous motor activity in the chick embryo. Previous findings have demonstrated that limb movements or kicks *in ovo* are driven by spontaneous electrical activity generated by spinal cord networks [39]–[40]. Therefore, the extent of activity blockade was assessed by counting the number of kicks (limb movements) generated by chicken embryos rather than by recording the electrical activity output from ventral roots [9]. The lack of spontaneous motor activity in infected embryos is not due to disruption of motoneuron survival, neurotransmitter expression or synapse formation. Thus, no changes in the number of *Islet*-positive neurons were reported at E10 following infection of chicken embryos with RCASBP(B)-Kir2.1, suggesting that inhibition of ongoing electrical activity could not be the result of a reduced number of functional motoneurons. Moreover, expression of Kir2.1 and blockade of ongoing electrical activity does not appear to interfere with the normal pattern of neurotransmitter expression. Thus, no changes in ChAT and GAD65 mRNA were detected in RCASBP(B)-Kir2.1-infected embryos. Although inhibition of ongoing electrical activity does not alter ChAT and GAD65 mRNA expression, we cannot rule out the possibility that other aspects of cholinergic and/or GABA-ergic synaptic neurotransmission in the spinal cord maybe affected by this procedure including changes in ChAT and GAD65 protein expression. Similarly, future work is needed to determine whether inhibition of ongoing electrical activity alters glutamatergic neurotransmission in the chicken spinal cord. Blockade of ongoing motor activity could also result from changes in the ability of axon terminals to make synaptic connections with muscle fibers. However, our present results indicate that blockade of electrical activity actually increases the number of synapses in one particular hindlimb muscle, the IFIB. Since synapse formation was investigated by double labeling of AChR cluster with presynaptic vesicles, we cannot ascertain whether those synapses are functional. There is also the possibility that the inhibitory effect of Kir2.1 on spontaneous motor activity could be the result of pathfinding errors involving the motoneuron axons. Previous results indicate that pharmacological blockade of spontaneous electrical activity alters the normal pattern of motor axon guidance by disrupting the expression of specific guidance molecules in the chicken [3], [15]. Since we did not characterize possible pathfinding errors in RCASBP(B)-Kir2.1-infected embryos, it is possible that changes in axonal projections to hindlimb target muscles could have altered spontaneous motor activity. Future work will determine whether chronic inhibition of spinal cord activity can result in pathfinding errors as previously reported in the case of pharmacological blockage of network activity [3], [15].

A reduction in the spontaneous motor activity is likely the result of changes in the electrical properties of motoneurons, particularly changes in the ability of motoneurons to respond to excitatory synaptic inputs. Infection of chicken embryos with RCASBP(B)-Kir2.1 caused a significant hyperpolarization of the resting membrane potential in motoneurons at all ages. There was also a significant decrease in the input resistance at E11. At E8, however, Kir2.1 expression did not result in a significant change in input resistance. Although the cause of this observation is unclear, it may reflect opposite changes in other channel conductances around resting membrane potential as a result of Kir2.1 expression [41]–[42]. Nonetheless, the overall changes in the electrical properties of motoneurons will reduce the ability of motoneurons to respond to depolarizing synaptic inputs. At all ages studied, however, motoneurons are still capable of generating action potentials. Although there was a significant age-dependent change in the action potential amplitude between E8 and E11, no significant changes were detected in RCASBP(B)-Kir2.1-infected embryos as compared with embryos infected with RCASBP(B).

During spinal cord development, approximately half of all neurons die as a result of programmed cell death by an apoptotic mechanism [43]–[44]. Although motoneuron survival depends on limited amounts of target-derived neurotrophic factors [45], activity-dependent changes in intracellular [Ca^2+^] may play a role in supporting cell survival. For example, Ca^2+^ influx generated by chronic membrane depolarization promotes survival of chicken lumbar motoneurons *in vitro* [46]. The survival-promoting effect of intracellular [Ca^2+^] is consistent with the Ca^2+^ set point hypothesis, which states that sustained elevations of intracellular [Ca^2+^] within a set range promote neuronal survival in the absence of trophic support [47]. However, a large increase in intracellular [Ca^2+^] will have a detrimental effect on cell survival [48]–[49]. There is considerable controversy regarding whether spontaneous electrical activity regulates motoneuron survival during normal development. *In ov*o studies using pharmacological blockers or direct electrical stimulation have failed to establish a conclusive link between spinal cord activity and motoneuron survival. For example, Usiak and Landmesser [13] reported that inhibition of ongoing spinal cord activity with muscimol does not alter motoneurons survival, whereas the opposite results were reported by Oppenheim et al. [14]. We should point out that although muscimol is a GABA agonist that blocks spontaneous electrical activity *in ov*o, GABA receptor activation evokes membrane depolarization and increased intracellular [Ca^2+^] in chicken spinal motoneurons at these developmental stages (present results & [50]). Thus, it is likely that muscimol may not only affect network activity but it may also alter other developmental processes by increasing intracellular [Ca^2+^]. In addition, direct electrical stimulation of chicken embryos *in ovo* has also failed to alter motoneuron survival [51].

Our present results indicate that blockade of ongoing activity in RCASBP(B)-Kir2.1-infected embryos did not result in any significant change in motoneuron survival at E10 as determined by assessing the number of *Islet*-positive neurons. Thus, we can conclude that inhibition of ongoing activity in the spinal cord does not alter motoneuron survival. Although we did not see an increase in motoneuron survival following inhibition of electrical activity under our experimental conditions, we detected a significant increase in synapse formation in RCASBP(B)-Kir2.1-infected embryos. Previous findings indicate that blockage of peripheral neuromuscular activity regulates synapse formation in chicken hindlimb muscles [28], [51]–[52]). Increased nerve branching appears to mediate the increase in synapse formation, which occurs following blockade of neuromuscular transmission. Our present results suggest that not only blockade of peripheral activity but also inhibition of centrally generated activity could result in increased synapse formation. Increased synapse formation in RCASBP(B)-Kir2.1-infected embryos could be mediated by either inhibition of network activity or due to Kir2.1 expression in individual motoneurons.

Expression of functional voltage-gated K^+^ channels plays a critical role in the regulation of neuronal excitability. For example, A-type and Ca^2+^-dependent K^+^ channels contribute to the repolarization of the action potential and can alter the rate of repetitive firing in neurons [53]. Previous studies have revealed that functional expression of Ca^2+^-dependent K^+^ channels is developmentally regulated in chicken spinal motoneurons [2]. Functional expression of Ca^2+^-dependent K^+^ channels is regulated in part by ongoing electrical activity in the chicken spinal cord and also by innervation of target tissue. Thus, chronic treatment of chicken embryos with the neuronal nicotinic acetylcholine receptor mecamylamine or the GABA receptor agonist muscimol results in a significant reduction in the current density of Ca^2+^-dependent K^+^ channels [2]. Similarly, ongoing electrical activity in the chicken spinal cord not only regulates the functional expression of A-type K^+^ channels but it also appears to regulate its inactivation kinetics [37]. Our present results are consistent with these previous findings. First, infection of chicken spinal cords with the RCASBP(B)-Kir2.1 construct reduces the functional expression of Ca^2+^-dependent K^+^ channels of E11 lumbar motoneurons. Downregulation of spinal cord activity also resulted in a significant reduction in the inactivation time constant of A-type K^+^ currents. An acceleration in the inactivation rate of A-type K^+^ currents could be construed as a compensatory response to an electrically depressed network, designed to potentially magnify any excitatory input in the motoneurons. Surprisingly, no change in the A-type K^+^ current amplitudes was found between control and RCASBP(B)-Kir2.1 infected embryos as previously reported by Casavant et al. [37]. In our study, activity was likely reduced from very early stages of development, whereas activity was only reduced between E6 and E10 in Casavant's work. Thus, it is possible that the length of activity inhibition may affect A-type K+ current amplitudes differently. There is also the possibility that Kir2.1 expression influences the expression of other endogenous ionic conductances such as A-type K^+^ channels. For example, overexpression of A-type K^+^ channels in pyloric neurons causes a significant increase in the endogenous expression of the hyperpolarization-activated inward current Ih [41]–[42].

Changes in K_Ca_ channel expression and A-current kinetics could be mediated by either a global effect of inhibiting spinal cord activity or just due to local changes in infected motoneurons. Although infection of chick spinal neurons with RCASBP(B)-Kir2.1 results in an infection rate of approximately half of all motoneurons, this leads to a considerable change in the K_Ca_ channel expression and A-current kinetics in the majority of recorded motoneurons. The present results suggest that these changes are most likely generated by changes in network activity rather than changes in the electrical properties of individual neurons as a result of Kir2.1 expression. For example, in chicken embryos infected with RCASBP(B)-Kir2.1 there is a significant change in the distribution of inactivation time constants of A-type K^+^ currents . In embryos infected with RCASBP(B), there is a widespread distribution of inactivation values, whereas Kir2.1 expression shifted the inactivation time constant to lower values. The argument that changes in K_Ca_ channel expression and A-current kinetics are the result of overall changes in network activity are further supported by previous work demonstrating similar effects of pharmacological blockade of spinal cord activity on channel expression *in vivo* and *in vitro* [2], [37]. The present results also suggest that peripheral changes in the level of activity at the neuromuscular junction are not responsible for changes in the electrical properties of motoneurons under our experimental conditions. It could be argued that increased synapse formation induced by blockade of electrical activity could explain the changes in K_Ca_ channel expression and A-current kinetics. However, we have previously reported that blockade of neuromuscular synaptic activity with tubocurare increases K_Ca_ expression [2], whereas A-currents are not affected at all by tubocurare–treatment of chicken embryos [37].

Ongoing electrical activity is an important regulatory mechanism of ion channel expression during neuronal development. In the chicken spinal cord, bursts of activity can be recorded in motoneurons before the establishment of synaptic connections with target muscles [8]. Changes in ion channel expression will in turn result in changes in the activation pattern of developing motoneurons. These changes could potentially regulate other developmental processes in the motoneurons including synapse elimination, axonal pathfinding and neuromuscular development [3], [5]–[6], [54]–[55]. Thus, activity-dependent changes in ion channel expression can provide a feedback mechanism that drives the overall maturation of the neuromuscular system.

## References

[1] Mellor JR, Merlo D, Jones A, Wisden W, Randall AD (1998). Mouse cerebellar granule cell differentiation: electrical activity regulates the GABAA receptor alpha 6 subunit gene.. J Neurosci..

[2] Martin-Caraballo M, Dryer SE (2002a). Activity- and target-dependent regulation of large-conductance Ca^2+^-activated K^+^ channels in developing chicken lumbar motoneurons.. J Neurosci..

[3] Hanson MG, Landmesser LT (2004). Normal patterns of spontaneous activity are required for correct motor axon guidance and the expression of specific guidance molecules.. Neuron.

[4] Borodinsky LN, Root CM, Cronin JA, Sann SB, Gu X (2004). Activity-dependent homeostatic specification of transmitter expression in embryonic neurons.. Nature.

[5] Phillips WD, Bennett MR (1987a). Elimination of distributed acetylcholine receptor clusters from developing fast-twitch fibres in an avian muscle.. J. Neurocytol.

[6] Phillips WD, Bennett MR (1987b). Elimination of distributed synaptic acetylycholine receptor clusters on developing avian fast-twitch muscle fibres accompanies loss of polyneuronal innervation.. J. Neurocytol.

[7] Jarvis JC, Sutherland H, Mayne CN, Gilroy SJ, Salmons S (1996). Induction of a fast-oxidative phenotype by chronic muscle stimulation: mechanical and biochemical studies.. Am J Physiol..

[8] Milner LD, Landmesser LT (1999). Cholinergic and GABAergic inputs drive patterned spontaneous motoneuron activity before target contact.. J Neurosci..

[9] O'Donovan MJ, Landmesser L (1987). The development of hindlimb motor activity studied in the isolated spinal cord of the chicken embryo.. J Neurosci..

[10] Hamburger V, Balanan M (1963). Observations and experiments on spontaneous rhythmical behavior in the chicken embryo.. Dev Biol..

[11] Ho S, O'Donovan MJ (1993). Regionalization and intersegmental coordination of rhythm-generating networks in the spinal cord of the chicken embryo.. J Neurosci..

[12] Chub N, O'Donovan MJ (1998). Blockade and recovery of spontaneous rhythmic activity after application of neurotransmitter antagonists to spinal networks of the chicken embryo.. J Neurosci..

[13] Usiak MF, Landmesser LT (1999). Neuromuscular activity blockade induced by muscimol and d-tubocurarine differentially affects the survival of embryonic chicken motoneurons.. J Neurosci..

[14] Oppenheim RW, Caldero J, Cuitat D, Esquerda J, Ayala V (2003). Rescue of developing spinal motoneurons from programmed cell death by the GABA(A) agonist muscimol acts by blockade of neuromuscular activity and increased intramuscular nerve branching.. Mol Cell Neurosci..

[15] Hanson MG, Landmesser LT (2006). Increasing the frequency of spontaneous rhythmic activity disrupts pool-specific axon fasciculation and pathfinding of embryonic spinal motoneurons.. J Neurosci..

[16] Gonzalez-Islas C, Wenner P (2006). Spontaneous network activity in the embryonic spinal cord regulates AMPAergic and GABAergic synaptic strength.. Neuron..

[17] Chen L, Yu YC, Zhao JW, Yang XL (2004). Inwardly rectifying potassium channels in rat retinal ganglion cells.. Eur J Neurosci..

[18] Dhamoon AS, Jalife J (2005). The inward rectifier current (IK1) controls cardiac excitability and is involved in arrhythmogenesis.. Heart Rhythm..

[19] Hollenbeck PJ, Fekete DM (2003). Expression of transgenes in primary neurons from chicken peripheral and central nervous systems by retroviral infection of early embryos.. Methods Cell Biol..

[20] Finn TP, Kim S, Nishi R (1998). Overexpression of ciliary neurotrophic factor in vivo rescues chicken ciliary ganglion neurons from cell death.. J Neurobiol..

[21] Sato N, Sakuma C, Kato H, Milligan CE, Oppenheim RW (2002). Bcl-2 rescues motoneurons from early cell death in the cervical spinal cord of the chickenen embryo.. J Neurobiol..

[22] Hughes SH (2004). The RCAS vector system.. Folia Biol (Praha)..

[23] Morgan BA, Fekete DM (1996). Manipulating gene expression with replication-competent retroviruses.. Methods Cell Biol..

[24] Johns DC, Marx R, Mains RE, O'Rourke B, Marban E (1999). Inducible genetic suppression of neuronal excitability.. J Neurosci..

[25] Hamburger V, Hamilton HL (1951). A series of normal stages in the development of the chicken embryo. 1951.. Dev Dyn..

[26] Hollyday M, Hamburger V (1977). An autoradiographic study of the formation of the lateral motor column in the chicken embryo.. Brain Res..

[27] Livak KJ, Schmittgen TD (2001). Analysis of relative gene expression data using real-time quantitative PCR and the 2(-Delta Delta C(T)) Method.. Methods.

[28] Dahm LM, Landmesser LT (1991). The regulation of synaptogenesis during normal development and following activity blockade.. J Neurosci..

[29] Martin-Caraballo M, Greer JJ (1998). Electrophysiological properties of rat phrenic motoneurons during perinatal development.. J Neurophysiol..

[30] Ni X, Sullivan GJ, Martin-Caraballo M (2007). Developmental characteristics of AMPA receptors in chick lumbar motoneurons.. Dev Neurobiol..

[31] Topert C, Doring F, Wischmeyer E, Karschin C, Brockhaus J (1998). Kir2.4: a novel K+ inward rectifier channel associated with motoneurons of cranial nerve nuclei.. J Neurosci..

[32] Ericson J, Thor S, Edlund T, Jessell TM, Yamada T (1992). Early stages of motor neuron differentiation revealed by expression of homeobox gene Islet-1.. Science.

[33] Caldero J, Prevette D, Mei X, Oakley RA, Li L (1998). Peripheral target regulation of the development and survival of spinal sensory and motor neurons in the chicken embryo.. J Neurosci..

[34] Tang J, Landmesser LT (1993). Reduction of intramuscular nerve branching and synaptogenesis is correlated with decreased motoneuron survival.. J Neurosci..

[35] Oppenheim RW, Prevette D, D'Costa A, Wang S, Houenou LJ (2002). Reduction of neuromuscular activity is required for the rescue of motoneurons from naturally occurring cell death by nicotinic-blocking agents.. J Neurosci..

[36] Chub N, Mentis GZ, O'Donovan MJ (2006). Chloride-sensitive MEQ fluorescence in chicken embryo motoneurons following manipulations of chloride and during spontaneous network activity.. J Neurophysiol..

[37] Casavant RH, Colbert CM, Dryer SE (2004). A-current expression is regulated by activity but not by target tissues in developing lumbar motoneurons of the chicken embryo.. J Neurophysiol..

[38] McCobb DP, Best PM, Beam KG (1990). The differentiation of excitability in embryonic chicken limb motoneurons.. J Neurosci.

[39] Landmesser LT, Szente M (1986). Activation patterns of embryonic chicken hind-limb muscles following blockade of activity and motoneurone cell death.. J Physiol..

[40] O'Donovan MJ, Chub N, Wenner P (1998). Mechanisms of spontaneous activity in developing spinal networks.. J Neurobiol..

[41] MacLean JN, Zhang Y, Goeritz ML, Casey R, Oliva R (2005). Activity-independent coregulation of IA and Ih in rhythmically active neurons.. J Neurophysiol..

[42] MacLean JN, Zhang Y, Johnson BR, Harris-Warrick RM (2003). Activity-independent homeostasis in rhythmically active neurons.. Neuron.

[43] Hamburger V (1975). Cell death in the development of the lateral motor column of the chicken embryo.. J Comp Neurol..

[44] Chu-Wang I-W, Oppenheim RW (1978). Cell death of motoneurons in the chicken embryo spinal cord. I. A light and electron microscopy study of naturally occurring and induced cell loss during development.. J Comp Neurol.

[45] Oppenheim RW (1989). The neurotrophic theory and naturally occurring motoneuron death.. Trends Neurosci..

[46] Soler RM, Egea J, Mintenig GM, Sanz-Rodriguez C, Iglesias M (1998). Calmodulin is involved in membrane depolarization-mediated survival of motoneurons by phosphatidylinositol-3 kinase- and MAPK-independent pathways.. J Neurosci..

[47] Johnson EM, Koike T, Franklin J (1992). A “calcium set-point hypothesis” of neuronal dependence on neurotrophic factor.. Exp Neurol..

[48] Tymianski M, Charlton MP, Carlen PL, Tator CH (1993). Source specificity of early calcium neurotoxicity in cultured embryonic spinal neurons.. J Neurosci..

[49] Caldero J, Ciutat D, Llado J, Castan E, Oppenheim RW (1997). Effects of excitatory amino acids on neuromuscular development in the chicken embryo.. J Comp Neurol..

[50] Chub N, O'Donovan MJ (2001). Post-episode depression of GABAergic transmission in spinal neurons of the chicken embryo.. J Neurophysiol..

[51] Fournier Le Ray C, Prevette D, Oppenheim RW, Fontaine-Perus J (1993). Interactions between spinal cord stimulation and activity blockade in the regulation of synaptogenesis and motoneuron survival in the chicken embryo.. J Neurobiol..

[52] Oppenheim RW, Prevette D, Houenou LJ, Pincon-Raymond M, Dimitriadou V (1997). Neuromuscular development in the avian paralytic mutant crooked neck dwarf (cn/cn): further evidence for the role of neuromuscular activity in motoneuron survival.. J Comp Neurol..

[53] Martin-Caraballo M, Greer JJ (2000). Development of potassium conductances in perinatal rat phrenic motoneurons.. J Neurophysiol.

[54] Hall BK, Herring SW (1990). Paralysis and growth of the musculoskeletal system in the embryonic chicken.. J Morphol..

[55] Persson M (1983). The role of movements in the development of sutural and diarthrodial joints tested by long-term paralysis of chicken embryos.. J Anat..

